# Selective Patch Angioplasty and Intraoperative Shunting in Carotid Endarterectomy: A Single-Center Review of 141 Procedures

**DOI:** 10.7759/cureus.367

**Published:** 2015-10-28

**Authors:** Rahul Kapoor, Alexander I Evins, Joshua Marcus, Luigi Rigante, Mayumi Kubota, Philip E Stieg

**Affiliations:** 1 Department of Neurological Surgery, Weill Cornell Medical College, New York Presbyterian Hospital, New York

**Keywords:** carotid, endarterectomy, selective patch, angioplasty, shunting, vascular

## Abstract

Objective: Open surgical treatment of carotid artery stenosis, namely, carotid endarterectomy (CEA), has evolved since its inception in 1953. Despite improvements in the treatment of carotid occlusive disease through technological and surgical innovations, the use of patch grafting in CEA’s remains controversial. We evaluate the durability of the primary closure and the safety of selective shunting during carotid endarterectomy (CEA) as determined by intraoperative EEG and postoperative outcomes.

Methods: A consecutive series of CEA’s performed by the senior author at a single academic medical center from 2001 to 2012 were reviewed. All cases were performed under continuous intraoperative electroencephalography (EEG). Patch angioplasty was used in cases where there was tortuosity of the vessel within the region of the endarterectomy and narrow vessel diameter at the distal end of the arteriotomy. Shunting was used when intraoperative EEG showed a > 50% reduction in a waveform in any lead. Patients were evaluated for restenosis via imaging or ultrasound at six months and subsequently annual follow-up.

Results: One hundred and forty-one CEA’s were performed on 132 (76 male, 56 female) patients with an average age of 71 years (range: 40–95 years). Four (3%) cases required patch angioplasty and three (2%) required intraoperative shunts. The cross-clamp time ranged from 22 to 74 minutes, and the duration increased with the use of shunts and patches. Complications were rare and included recurrent stenosis (n=2), postoperative transient ischemic attack (n=1), ischemic stroke in (n=1), temporary hypoglossal nerve weakness (n=2), temporary marginal mandibular nerve weakness (n=6), and neck hematoma (n=1).

Conclusion: Intraoperative EEG data suggests that primary closure and selective shunting in CEA can result in outcomes comparable with routine patch angioplasty and shunting.

## Introduction

Open surgical treatment of carotid artery stenosis, namely, carotid endarterectomy (CEA), has evolved since its inception in 1953 [1-2]. Despite improvements in the treatment of carotid occlusive disease through technological and surgical innovations, the use of patch grafting in CEA’s remains controversial. A number of studies have suggested a lower risk of postoperative restenosis with the use of both patch angioplasty and primary closure during CEA’s [3-9], although other studies have shown that the occurrence of restenosis may be independent of primary or patch closure [10]. Similar disagreement also surrounds the use of temporary shunts in CEA’s. While some surgeons prefer routine shunting, other surgeons opt for selective shunting during surgery [11]. Primary closure versus patch angioplasty and selective versus routine shunting during CEA’s have their respective risks and benefits, and patient outcomes should be used to evaluate the benefits and risks associated with different surgical techniques [12-14], including rate of restenosis, infection, myocardial infarction (MI), urinary tract infection (UTI), cerebrovascular accidents, and transient ischemic attacks (TIA). We review 141 CEA’s performed at our institution between 2001 and 2012 and evaluate the use of primary closure and selective shunting.

## Materials and methods

Informed patient consent was obtained at the time of treatment. No identifying patient information is contained within this study.

### Patient selection

A consecutive series of CEA’s performed by the senior author at a single academic medical center from 2001 to 2012 were reviewed. Initial diagnoses, surgical procedure, and recovery were evaluated, along with patient demographics (age, gender, and ethnicity), diagnostic characteristics (severity and side of occlusive disease), and preexisting co-morbidities. Body Mass Index (BMI) was calculated for each patient; patients with a BMI between 25.0 and 29.9 were classified as overweight and those with a BMI of 30.0 or greater as obese. Carotid stenosis was defined as symptomatic if the patient had a history of an ipsilateral ischemic stroke or TIA within one year preceding the CEA. Operative data, including EEG, shunt/patch usage, clamp time, and operative time was also evaluated. 

### Preoperative evaluation

At least two radiological exams, including carotid ultrasound, magnetic resonance angiography (MRA), computerized tomography angiography (CTA), and/or catheter angiography, were performed in order to confirm the presence and severity of carotid artery stenosis. Additionally, all patients underwent cardiovascular and neurological exams prior to surgery and, in a number of cases, were treated with statins and antiplatelet medications as needed.

### Surgical procedure

**Figure 1:**
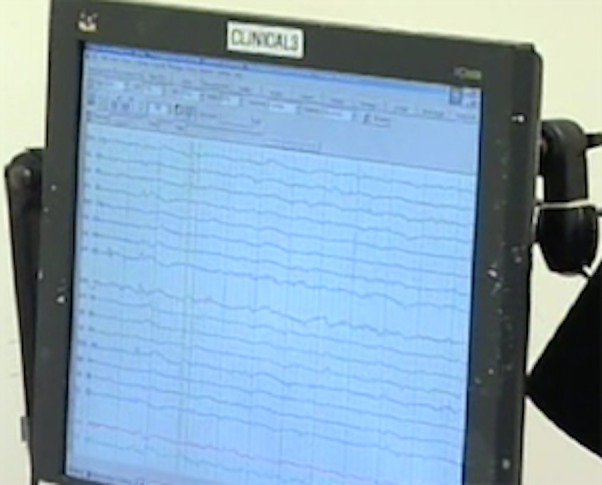
Intraoperative Electroencephalography In all cases, CEA was performed under continuous intraoperative EEG.

CEA was performed under general anesthesia with continuous intraoperative electroencephalography (EEG) (Figure 1).

**Figure 2:**
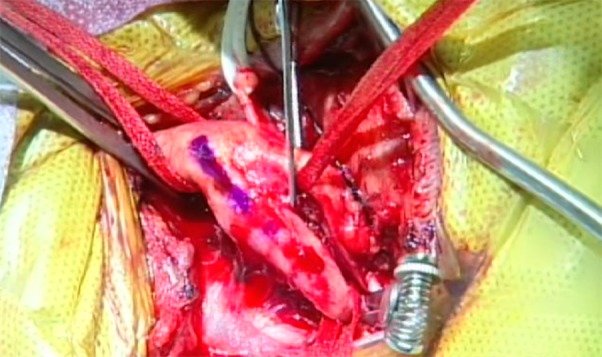
Clamping of the Distal Internal Carotid Artery The distal internal carotid clamp was placed sufficiently distal to the plaque in order to avoid induction of an embolic event.

All patients received 5,000 units of heparin 3 minutes prior to cross-clamping. The distal internal carotid clamp was placed sufficiently distal to the plaque to avoid induction of an embolic event, and each patient’s systolic blood pressure was elevated to 180 mmHg for all patients (Figure 2).

**Figure 3:**
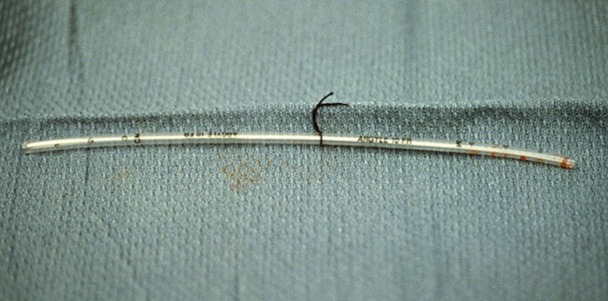
Argyle Shunt If a shunt was required, an Argyle shunt was placed distally into the internal carotid artery and, after back bleeding, was placed proximally in the common carotid artery and secured with Rommel tourniquets to prevent bleeding.

If a shunt was required, an Argyle shunt was placed distally into the internal carotid artery and, after back bleeding, was placed proximally in the common carotid artery and secured with Rommel tourniquets to prevent bleeding (Figure 3).

**Figure 4:**
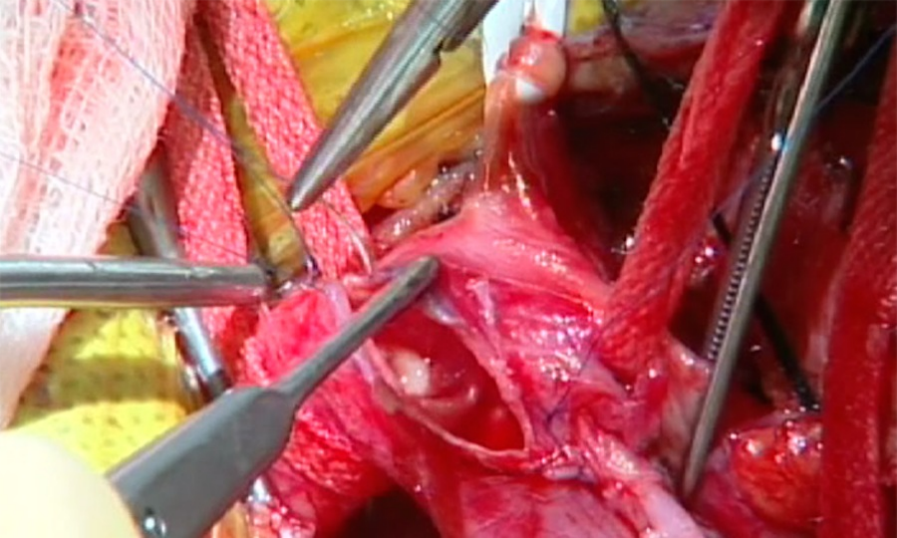
Primary Closure Primary closure was performed with 6–0 prolypropylene sutures running from the distal to the proximal end of the vessel.

**Figure 5:**
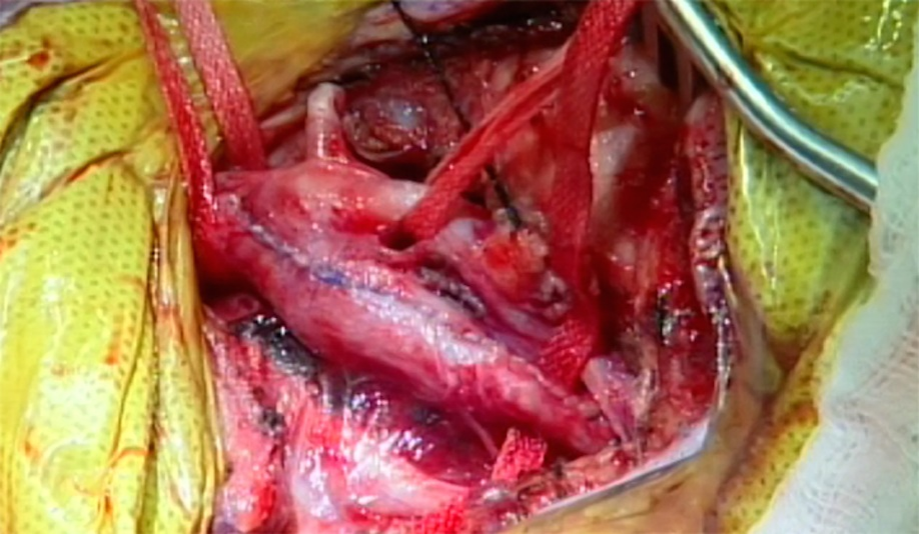
Primary Closure Watertight closure was achieved with primary closure.

Primary closure was performed with 6–0 prolypropylene suture (PROLENE^®^, Ethicon Inc., Somerville, NJ) running from the distal to the proximal end (Figures 4-5).

**Figure 6:**
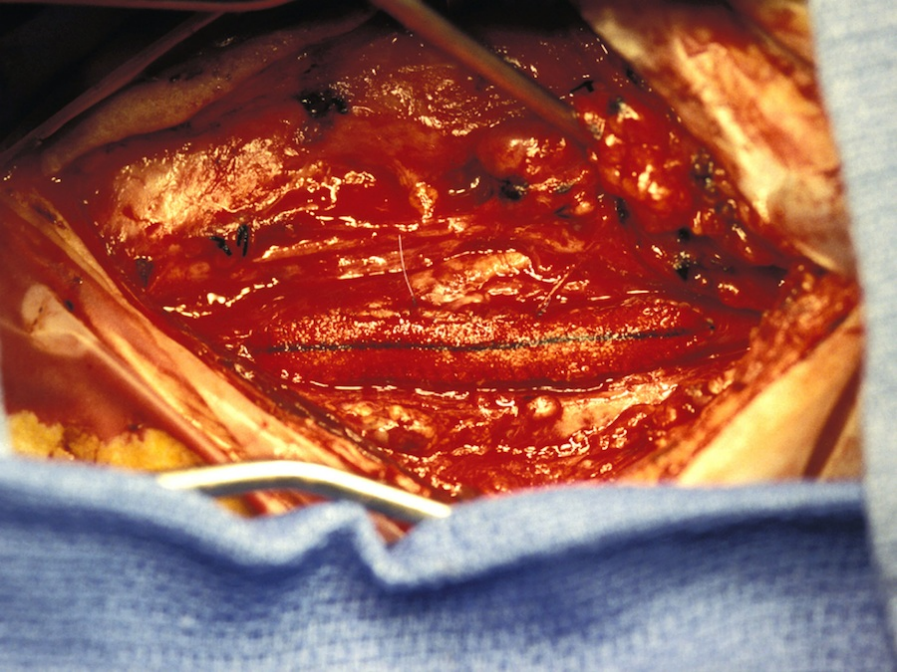
Patch Angioplasty If patch angioplasty was required, a Dacron patch was configured and sutured into position using 6-0 prolypropylene sutures.

If a patch was required, a Dacron patch (Hemashield™, Atrium Medical Inc., Hudson, NH) was configured and sutured into position using a 6-0 polypropylene suture (Figure 6).

### Postoperative course and follow-up

Patients were discharged within 24 hours unless cardiac follow-up was required. All patients were seen at follow-up by the surgeon and neurologist at two weeks and thence every three months for one year, and annually thereafter. The patients were evaluated for restenosis via imaging or ultrasound at six months and subsequently annual follow-up. All patients received antiplatelet medication and statins postoperatively as dictated by their medical co-morbidities.

## Results

One hundred and forty-one CEA’s were performed on 132 (76 male, 56 female) patients with an average age of 71 years (range: 40–95 years) at our institution between 2001 and 2012 (Table 1). Of the patients whose race and ethnicity were reported, there were 111 Caucasians, 5 African Americans, 5 Asians, and 11 Hispanics.

**Table 1:** Patient Characteristics

Mean Age at Surgery	71 ± 9.2 years
Sex	n (%)
Male	76 (58%)
Female	56 (42%)
Ethnicity	n (%)
Caucasian	111 (84%)
African American	5 (4%)
Asian	5 (4%)
Hispanic	11 (8%)
Co-morbidities	n (%)
Hypertension	107 (81%)
Hyperlipidemia	102 (77%)
Heart disease	66 (50%)
Coronary artery disease	43 (33%)
Atrial fibrillation	6 (4.5%)
Smoking	78 (59%)
Diabetes mellitus	33 (25%)
Obesity/overweight	68 (52%)
Multiple co-morbidities	117 (89%)

Co-morbidities included hypertension in 107 (81%) patients, hyperlipidemia in 102 (72%), cigarette smoking in 78 (59%), obesity in 36 (27%), and 32 (23%) were classified as overweight. Seventy-one (50.4%) cases were symptomatic.

Seventy-nine (56%) CEA’s were performed on the right and 63 (44%) on the left, with nine (6.4%) cases performed bilaterally. Stenosis was considered severe, according to the NASCET Criteria, in 118 (84%) cases (with > 70% narrowing) and moderate (50–70% narrowing) in the remaining 23 (16%) patients (Table 2).

**Table 2:** Carotid Endarterectomy (CEA) Cases

Type	n (%)
Bilateral cases	9 (6%)
Symptomatic	71 (50%)
Severe stenosis	118 (84%)
Moderate stenosis	23 (16%)
Right CEA	79 (56%)
Left CEA	63 (45%)
Shunt	3 (2%)
Patch angioplasty	4 (3%)
Primary closure	137 (97%)

Only four (3%) cases required patch angioplasty and three (2%) required intraoperative shunts. The cross-clamp time ranged from 22 to 74 minutes, and the duration increased with the use of shunts and patches (Table 3). Only two (1.4%) of cases were noted to have recurrent stenosis.

**Table 3:** Surgical Complications

Major Complications	
Recurrent stenosis	3 (2.1%)
Transient ischemic attack	1 (0.7%)
Stroke	1 (0.7%)
Minor Complications	
Hypoglossal nerve weakness	2 (1.4%)
Marginal mandibular nerve weakness	6 (4.3%)
Hematoma	1 (0.7%)

Other associated complications included one case of postoperative TIA, one case of ischemic stroke, two cases of temporary hypoglossal nerve weakness, six cases of temporary marginal mandibular nerve weakness, and one case of neck hematoma. No myocardial infarctions, urinary tract infections, or wound dehiscence were noted postoperatively.

## Discussion

### Primary closure and carotid patching

The use of patch angioplasty, as opposed to primary closure, in CEA’s remains controversial and is based primarily on the surgeon’s preference and experience. The data presented herein suggests that primary closure can result in successful patient outcomes while avoiding the risks associated with patch angioplasty, namely, increased cross-clamp time that may cause a greater neurocognitive decline, especially in the elderly population [9]. Other risks include needle hole bleeding, rupture, infection, and pseudoaneurysms. Some cases, however, still require patch angioplasty.

Autologous vein and synthetic patches are most commonly used during CEA’s [15], though increased cross-clamp time for vein harvesting, most commonly of the saphenous vein [15], is associated with the use of an autologous graft. Harvesting a vein also requires a second incision and the incidence of rupture (reported as 0.5-4%) of the saphenous vein patch is higher than that of synthetic grafts [16-18]. Patch rupture or bleeding, though rare, requires further intervention with incremental time, morbidity, and cost, although vein patches do provide a lower risk of infection and bleeding through the suture hole and are more resistant to thrombosis by providing an endothelial cell surface [15].

Polytetrafluoroethylene (PTFE) and Dacron patches are the most commonly used synthetic materials for patch angioplasty [15]. Similar patient outcomes have been observed with use of either material [19-20], although Dacron patches were preferred at our institution for cases that required patch angioplasty as they are easily deformed and less likely to produce needle hole bleeding. Dacron patches are, in our opinion, pliable and easier to handle during closure of CEA’s. Patch thickness is very amenable to passing needles during closure of the arteriotomy [21-22].

### Parameters for patch angioplasty

Based on our experience, primary closure of arteriotomies during CEA can lead to favorable patient outcomes while avoiding the risks associated with patch angioplasty. The data we report suggests that patch angioplasty is best applied to specific cases and may lead to successful patient outcomes by reducing the risk of restenosis. The specific guideline used for patching at our institution included severe tortuosity near the region of the arteriotomy and narrowing of the distal internal carotid beyond the arteriotomy of less than 2 mm, usually seen in patients with a string sign noted preoperatively [9].

### Routine and selective carotid shunting

Routine use of shunts during CEA’s is another issue that is debated in the literature [2, 23]. While some surgeons prefer routine shunting during CEA’s, others choose to completely avoid or selectively use shunts. Studies that support routine shunting show that shunts play an important role in reducing the risk of intraoperative and postoperative stroke [11, 24-26]. However, studies that support selective shunting also highlight a lower risk of stroke, further complicating clinical decision making on its use [27].

We believe that selective shunting during CEA’s, as determined by significant changes on EEG—a reliable tool for indirectly monitoring changes in cerebral blood flow [27-29]—leads to successful patient outcomes while avoiding the risks associated with routine shunting, namely, distal embolic complications during placement of the shunt [30]. Other risks include increased operative time, vessel dissection, incomplete endarterectomy, and cost of the CEA.

### Parameters for using shunts

A change in EEG during CEA might indicate that cerebral blood flow from contralateral circulation is not providing sufficient perfusion and thus indicates that a shunt is required to restore proper perfusion, especially when the average blood pressure remains constant during EEG fluctuations. Reduction in higher frequency waves, namely alpha and beta waves, are indicative of cerebral ischemia during CEA’s [27-29, 31-32]. At our institution, a preoperative baseline for EEG activity is established for each patient undergoing CEA. A significant reduction, marked by a 50% decrease in the amplitude of high-frequency waves compared to baseline, in any lead during the procedure is interpreted as insufficient blood flow from the contralateral side and warrants the use of a shunt in order to prevent cerebral ischemia.

### Postoperative complications

Adverse outcomes were rare in our series, and only two patients developed recurrent carotid stenosis. In the first case, a 69-year-old male, who initially presented with an asymptomatic severe carotid stenosis and underwent an uneventful CEA, developed severe recurrent stenosis at one-year follow-up. However, close examination of the patient’s studies revealed that the stenosis was not atherosclerotic in nature, but rather stemmed from the tortuosity of the distal end of the repair and myointimal hyperplasia. The patient also had several comorbidities, including heart disease, hyperlipidemia, and hypertension. The patient successfully underwent carotid artery stenting for recanalization of the artery. The second patient, a 76-year-old woman with symptomatic severe carotid occlusive disease, hypertension, and hyperlipidemia developed severe recurrent stenosis one year postoperatively. The patient did not have patch angioplasty per our protocol and her restenosis was secondary to myointimal hyperplasia.

Additional complications, including vessel occlusion, TIA, ischemic stroke, and hematoma, were noted. A 60-year-old man with a history of high cholesterol and diabetes mellitus had asymptomatic complete occlusion of the left internal carotid artery distal to the endarterectomy site one-year post-CEA, even though a patch was used to increase the initial diameter of the artery. A 73-year-old female, who initially presented with symptomatic disease and had a history of diabetes mellitus, suffered a TIA several days postoperatively. Postoperative CTA showed no stenosis along the arteriotomy and MRI showed no acute stroke. The patient was treated with aspirin and has had no further episodes. A 65-year-old man with hyperlipidemia suffered a small ischemic stroke after CEA possibly due to a tandem lesion. This patient initially presented with bilateral carotid occlusive disease and elected to undergo CEA on the contralateral side two months after the postoperative stroke without complication. A 49-year-old man with a history of smoking, hypertension, and hyperlipidemia suffered a postoperative hematoma, although no active bleeding site was identified on re-exploration.

### The role of statins

The role of medical management, particularly statins, in the treatment of cerebrovascular diseases has reduced the long-term morbidity and mortality associated with CEA. Additionally, statins show several short-term pleiotropic benefits, including vasodilation, atherosclerotic plaque stabilization, and thrombotic inhibition. These benefits have been shown to improve perioperative and long-term neurological outcomes, including survival  [33-38]. However, the role of statins in treating recurrent stenosis is still unclear. The rate of recurrent stenosis after CEA has been reported in the literature from 1% to 36%. As reported herein, the rate in this series is less than 2%. Moreover, the majority of recurrent lesions occur during the early postoperative period (< 3 years) and usually near the arteriotomy site, suggesting that myointimal hyperplasia is the primary cause of stenosis and not an atherosclerotic plaque. This is consistent with the release of platelet-derived growth factor and fibroblast growth factor by smooth muscle cells, endothelial cells, and platelets that contribute to smooth muscle proliferation at the incision site [39-42]. The role of statins in inhibiting this process is unclear [39, 43-44]. Several animal models have suggested that statins, especially simvastatin, inhibit the proliferation of smooth muscle cells, although human clinical trials have not found such a link [39]. Therefore, statins do not currently seem to play a role in the treatment of recurrent stenosis during the early postoperative period.

### Carotid ultrasonography

Even though several efficient imaging modalities exist, including catheter angiography, CTA, MRA, and carotid ultrasound, to evaluate the severity of stenosis, cost-effectiveness has become a significant factor in the clinical selection of suitable imaging techniques. Traditional angiography provides the most accurate assessment of carotid stenosis, although the procedure is associated with a significantly higher risk of stroke (0.5%) when compared to other imaging modalities [45-47]. While angiography is also the least cost-effective method, carotid ultrasound is the most cost-effective means of evaluating stenosis, especially during the postoperative period [47]. Ultrasound also has high accuracy and efficacy for evaluating stenosis along with a lower risk of complications when compared to traditional angiography [47]. For the patients in this series, the utility and cost-effectiveness of carotid ultrasounds have promoted their use in the postoperative care of patients who underwent CEA. When severe recurrent stenosis is suggested by ultrasound, a supplementary CTA or MRA should be obtained.

## Conclusions

The use of patch angioplasty and shunts during CEA remains controversial and their use remains dependent on the preference and experience of the surgeon. There is currently insufficient data in the literature on the risks and benefits associated with routine patch angioplasty and shunts during CEA. This retrospective series demonstrates that primary closure and selective shunting in CEA, based on intraoperative EEG monitoring, can result in outcomes comparable with routine use of patch angioplasty and shunts.
